# Finding Solvable Units of Variables in Nonlinear ODEs of ECM Degradation Pathway Network

**DOI:** 10.1155/2017/5924270

**Published:** 2017-05-30

**Authors:** Shuji Kawasaki, Dhisa Minerva, Keiko Itano, Takashi Suzuki

**Affiliations:** ^1^Iwate University, Morioka 020-8550, Japan; ^2^Osaka University, Toyonaka 560-8531, Japan

## Abstract

We consider ordinary differential equation (ODE) model for a pathway network that arises in extracellular matrix (ECM) degradation. For solving the ODEs, we propose applying the mass conservation law (MCL), together with a stoichiometry called* doubling rule*, to them. Then it leads to extracting new units of variables in the ODEs that can be solved explicitly, at least in principle. The simulation results for the ODE solutions show that the numerical solutions are indeed in good accord with theoretical solutions and satisfy the MALs.

## 1. Introduction

Differential equations are a useful method for modeling dynamics of reaction pathways in cells. They can be used to formulate biochemical kinetics, that is, the interactive dynamics of systems consisting of proteins, enzymes, products, and other components in terms of their transitive concentrations.

The biochemical kinetics of a system describes the reactions through the mass action law (MAL); this results in a system of first-order nonlinear ordinary differential equations (ODEs). In general, the nonlinear ODEs cannot be solved explicitly, and so they are usually studied by numerical simulations.

For the modeling of such a system, we propose an idea of using the mass conservation laws (MCLs) together with the MALs. That is, the variables in the ODEs obtained by the MALs are grouped into new units of variables to constitute new MALs, according to local balancing relations of inflows and outflows. It is important here that these new MCLs hold for all *t* ≥ 0. As a result, the nonlinear ODEs presented by the new units of variables turns out to be a* completely integrable system*; that is, the ODEs can be solved explicitly, at least in principle.

We apply this idea to analyze the kinetics of the molecule concentration dynamics in cancer cell invasion to the ECM by a matrix metalloproteinase called MT1-MMP. If we can find appropriate MCLs for balancing relations in the kinetics, then such relations turn out to exhibit linear relations between ODE variables that are valid for all *t* ≥ 0. Also, the nonlinear ODEs with the new unit of variables are completely solvable explicitly. That is, these nonlinear ODEs of new unit of variables can be solved* as a group*. These groups may often correspond to network motifs, that is, local functions; they suggest a bundle of meaningful components in a complex pathway network (PWN). As the simulation results show, the numerical solutions are in good accord with the theoretical solutions, indicating our modeling by the new unit of variables is right.

We thus clarify how to look at the PWN model of the ECM degradation with the unit variables. In fact, we indicate that the relevant system of the PWN can be grouped into several units of original variables and, with the unit variables, the system of ODEs can be solved explicitly. Taking linear combination of ODEs for the grouping has existed so far, but changing original ODEs into new, solvable ones by the linear combination has not been considered so far. This is a special stoichiometry. In addition, in order to enjoy such grouping method, certain reformulation including* doubling rule* is necessary as in the following, which has not been considered properly so far.

The organization of the paper is as follows.  Section 2 discusses four elementary reaction processes that are modeled by second-order nonlinear ODEs that can be solved explicitly. In Section 3, the ECM degradation mechanism is introduced, and we present one of the key concepts behind the choice of a good model for ODE kinetics, the* doubling rule* in stoichiometry. In Section 4, as an application of elementary reaction processes, we consider solving the ODE system that arises from the PWN of molecule concentration dynamics in ECM degradation. We list the tables cited in Section 4 and present the explicit solutions to the ODE system in Tables 1–7 and Appendix A, respectively.

## 2. Elementary Reaction Processes That Reduce to Solvable Second-Order Riccati Equations

In this section, we will show how the ODEs for the elementary reaction processes can be solved by reduction to the well-known Riccati equations of a new unit of variables.

In PWN, in addition to the simple dimerization of monomers, there are polymerizations of higher complexes. For those complexes that are modified from relevant monomers, we will assume that the association and dissociation constants are the same as those for the monomer associations or dimer dissociations, respectively.

For simplicity, we will show the four basic forms of elementary reactions as in the following. The association dissociation constants are here denoted by *k* and *l*, respectively.

(i) Association of two different molecules/dissociation of their product:
  (1) $$\begin{array}{c} A+B\overset{k}{\underset{l}{⇌}}AB. \end{array}$$
(ii) Association of the same molecules/dissociation of their product:
  (2) $$\begin{array}{c} A+A\overset{k}{\underset{l}{⇌}}AA. \end{array}$$
(iii) Association with a modified molecule/dissociation of their product:
  (3) $$\begin{array}{c} A+AB\overset{k}{\underset{l}{⇌}}ABA\;\;or\;\;AAB. \end{array}$$
(iv) Association with a homodimer/dissociation of their product:
  (4) $$\begin{array}{c} A+BB\overset{k}{\underset{l}{⇌}}ABB. \end{array}$$

Note that $AB+B\overset{k}{\underset{l}{⇌}}ABB$ is the same as reaction (iii).

We now show that the ODEs for the MALs for reactions (i)–(iv) can be solved explicitly. First, the MALs for reaction (i) are

(5) dAdt=−kAB+lP,dBdt=−kAB+lP,dPdt=kAB−lP.

The resulting system of ODEs can be solved explicitly: (5) implies the MCLs

(6a) dA+Pdt=dB+Pdt=0⟺A+P≡IA,B+P≡IB,

(6b) dA−Bdt=0⟺A−B=IA−IB

for some positive constants *I*_*A*_ and *I*_*B*_, which are typically the initial values of [*A*]+[*P*] and [*B*]+[*P*], respectively. Then, substituting

(7) A=IA−P,B=IB−P

into the third equation in (5), we obtain an equation in only the variable [*P*]:

(8) dPdtkIA−PIB−P−lP=kP2−kIA+IB+lP+kIAIB≡kP−z1P−z2,

where the last expression is the factorization of the quadratic polynomial in the second line. If *z*_1_ ≠ *z*_2_, this leads to

(9) $$\begin{array}{c} \left(\;\frac{1}{\left[P\right]-z_{2}}-\frac{1}{\left[P\right]-z_{1}}\;\right)\frac{d\left[P\right]}{dt}=-k\left(\;z_{1}-z_{2}\;\right), \end{array}$$

or ([*P*] − *z*_1_)/([*P*] − *z*_2_) = *Ce*^−*k*(*z*_1_−*z*_2_)*t*^, and hence the explicit solution

(10) Pt=z1−Cz2e−kz1−z2t1−Ce−kz1−z2t=z2+z1−z21−Ce−kz1−z2t,C=P0−z1P0−z2

for *t* ≥ 0. Here, *z*_1_, *z*_2_ are

(11) z1,z2=kIA+IB+l±kIA+IB+l2−4k2IAIB2k.

If *z*_1_ and *z*_2_ are distinct and *z*_1_ − *z*_2_ > 0 so that lim_*t*→*∞*_[*P*]_*t*_ = *z*_1_ in (10), then we find that [*P*]_*t*_ is monotonically decreasing and nonnegative. Solutions to [*A*] and [*B*] can be found in a similar manner by using (7). Although the situation is slightly different, a similar calculation was presented in ([1], Section  6.5).

Second, for reaction (ii), the MAL yields kinetic equations of the form

(12) dAdt=−2kA2+2lP,dPdt=kA2−lP.

In the first equation of (12), the coefficient of 2 stems from the fact that, in the reaction *A* + *A* → *P*, two molecules of *A* are consumed; similarly, in the dissociation, two molecules of *A* are produced. This is called a* double rate of consumption/reproduction.* The ODEs (12) imply the MCL

(13) dA+2Pdt=0⟺A+2P≡IA

for a positive constant *I*_*A*_. Then, substituting 2[*P*] = *I*_*A*_ − [*A*] into the first equation of (12), we have

(14) dAdt−2kA2−lA+lIA≡−2kA−ζ1A−ζ2,Atζ1−Cζ2e−2kζ1−ζ2t1−Ce−2kζ1−ζ2t=ζ2+ζ1−ζ21−Ce−2kζ1−ζ2t,C=A0−ζ1A0−ζ2

for *t* ≥ 0. Here *ζ*_1_, *ζ*_2_ are

(15) $$\begin{array}{c} \zeta_{1},\zeta_{2}=\frac{-l\pm \sqrt{l^{2}+8klI_{A}}}{4k}. \end{array}$$

Since *ζ*_1_ − *ζ*_2_ > 0, we have lim_*t*→*∞*_[*A*]_*t*_ = *ζ*_1_. As in case (i), the solution is monotonically decreasing and nonnegative. The solution to [*P*] can also be obtained by the MCL (13).

For reaction (iii), we have the MALs and MCL as shown in the following:

(16) dAdt=−kAAB+lP,dPdt=kAAB−lP,

(17a) dA+Pdt=0,

(17b) dA−ABdt=0.

The ODEs (16) can be solved in a manner similar to that used for reactions (i) and (ii). In fact, the association of *A*-*BA* reduces to reaction (i), and the association of *A*-*AB* reduces to reaction (ii).

Finally, for reaction (iv), we have the following MALs and the MCL:

(17) dAdt=−2kABB+lABB,

(18) dA−BBdt=0,dA+ABBdt=0.

The coefficient of 2 in 17 arises because a molecule of *A* may bind to either of the two *B* molecules. The solution to these ODEs 17 is the same as that for reaction (i) but with *k* replaced by 2*k*. This is called a* double chance of association*.

These elementary reaction processes (i)–(iv) will be used below to solve the ODE system for the kinetics of ECM degradation. The given ODE system is grouped into several subpathways, and in the reformulation based on these groups, we will find that the new variables reduce the equations* as groups* to those of the elementary reaction processes.

## 3. Application to the Model of ECM Degradation by MT1-MMP

In this section, we show how the elementary reaction processes in the previous section can be applied to a problem in cell biology, that of extracellular matrix (ECM) degradation by membrane type 1 matrix metalloproteinase (MT1-MMP), which is a step in the progression of cancer.

### 3.1. ECM Degradation Mechanism

As is well known, in order for the proliferated cancer cells to metastasize from a primary lesion, they first invade and degrade the ECM and then migrate. The cancer cells secrete MMP, and its dimer MMP2 is then activated by MT1-MMP; the MMP2 then degrades the ECM.

After the ECM is degraded, other enzymes, including MT1-MMP, begin degrading the interstitium beyond the ECM. Therefore, mathematical or biological elucidation of the activation process of MMP2 is important for finding a clinical cure or developing drugs. Below, we will denote MMP2, TIMP2, and MT1-MMP by *a*, *b*, and *c*, respectively.

**Figure 1:**
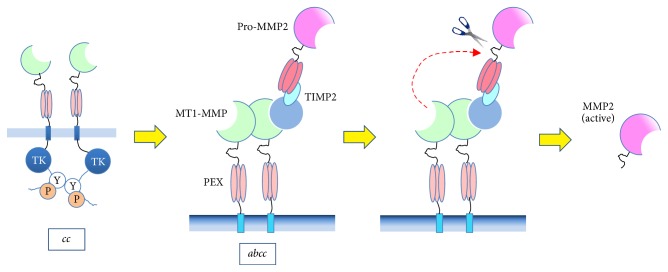
Mechanism of activation of* a* (MMP2) by* b* (TIMP2) and* c* (MT1-MMP).

Sato et al. [2] have revealed experimentally the mechanism of the activation of MMP2. The steps of the activation scenario of MMP2, as a cell biological model, proceed as follows (see Figure 1):

(0) Although *c* (MT1-MMP) is an ECM degradation enzyme as well, it is usually inactivated by *b* (TIMP2) immediately after vesicle transportation to the cell membranes. However, *c* activates *a* (MMP2), which is another ECM degradation enzyme, as follows.
(1) The *c* molecules that penetrate the cell membrane form homodimers *cc* on the membrane.
(2) On one of the *c* in the dimer -*cc*-, the heterodimer *ab* (pro-MMP2, TIMP2) is coupled to produce the tetramer *abcc*; alternatively, *a*- may be coupled with the heterotrimer -*bcc* to produce the tetramer. Thus, *a* is associated with *cc* via *b*.
(3) One of the *c* in *abcc* that is not coupled with *ab* cuts the connection between *a* and *b*.
(4) *a* that was cut and released then becomes the activated one.

This process is thus the associations/dissociations of the three molecules *a*, *b*, and *c*, and *abcc* is the key molecule in the activation of *a*. In order to produce certain amount of activated *a* (MMP2), there must be sufficient *b*. However, if there is too much *b*, then the remaining vacant site of *abcc*- tends to associate with -*b*, which results in the fact that *abccb* or *abccba* will be produced preferentially to *abcc*, and an insignificant amount of *a* will be activated. See Hoshino et al. [3] and then Watanabe et al. [4].

**Figure 2:**
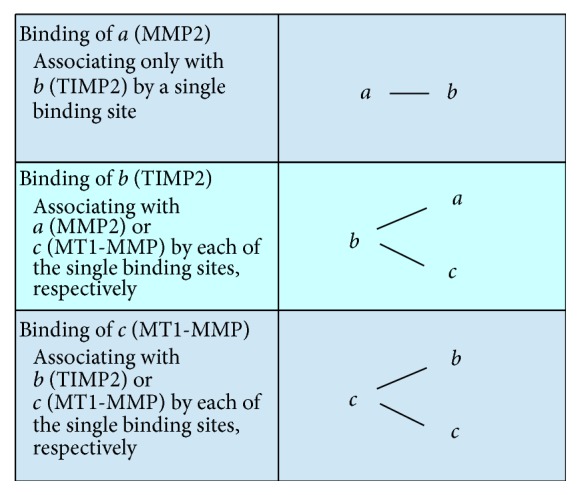
Binding sites of* a* (MMP2),* b* (TIMP2), and* c* (MT1-MMP).

The molecular binding rules that follow from this scenario are that *a* and *c* do not couple with each other, but with *b*, and *c* may form the homodimer *cc* (see Figure 2). Although it is difficult to deny the possibility of other binding patterns, to the best of our knowledge, there is no evidence of such patterns in the literature. Therefore, we will assume the following binding patterns:

1. *a* has a site of binding only with *b*.
2. *b* has a site of binding with *a* and another site of binding with *c*.
3. *c* has a site of binding with *b* and another site of binding with *c* itself.
4. There are no other binding patterns.

**Figure 3:**
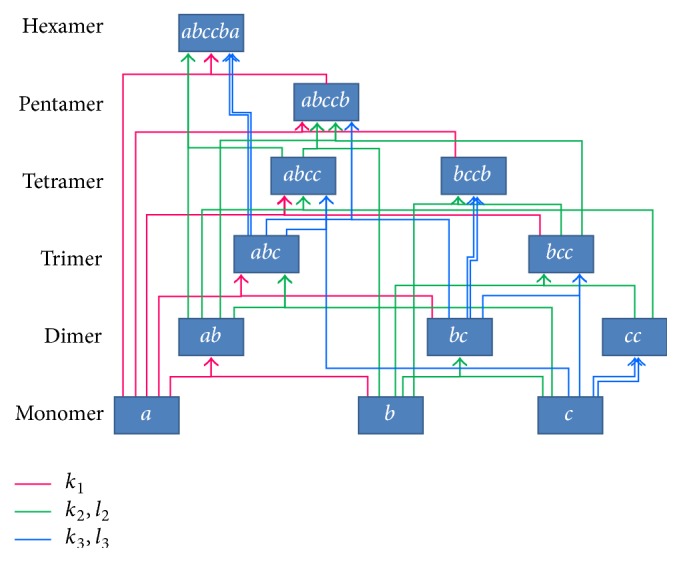
Pathway network of association/dissociation pairs of complexes of* a* (MMP2),* b* (TIMP2), and* c* (MT1-MMP). Here, arrows indicating only association are depicted.

By inspection, we see that there appear to be nine complexes (dimers to hexamer) obtainable from the monomers *a*, *b*, and *c*. The PWN of association or dissociation of these complexes are shown in Figure 3.

Thus, a quantitative model of ECM degradation can be based on the kinetics of associations/dissociations of the three proteins (*a*: MMP2; *b*: TIMP2; and *c*: MT1-MMP) and their nine complexes, for a total of twelve compounds. The PWNs of the twelve molecules are depicted in Figure 3. A quantitative model was first considered by Karagiannis and Popel [5], in which the general interactive behavior of the molecules *a*, *b*, and *c* was investigated through simulation in the context of type-I collagen proteolysis. Further, their behavior in more realistic situations was considered by Hoshino et al. [3] and then by Watanabe et al. [4] as the mathematical interpretation of the cell biological model in [2].

In the quantitative models in [3, 4], the detailed cell biological behaviors of these enzymes (*a*, *b*, and *c*) were studied. In particular, they found that MT1-MMP has two fluorescence recovery time constants, 29 [s] and 259 [s], and, in FRAP experiments, they determined that the recovery is not due to lateral diffusions but to the insertion of a new membrane. As a result, they verified by simulation that the inactivation of MT1-MMP by TIMP2 proceeds very quickly (halving time is 4.5 [s]); therefore, a very rapid turnover of MT1-MMP must be occurring at the location of the ECM degradation.

We consider a mathematical treatment of the ODE kinetics with a more complete theoretical analysis of such quantitative studies; see E1–E12.

Now, according to the binding rules discussed above, we have the following three associations or dissociations in the PWN:

(I) Association of *a*-*bB* (association constant *k*_1_):
  (19) a-b a+bB⇌l1≡0k1abB.
  The molecules *a* and *b* do not dissociate after they are once associated. Thus we set *l*_1_ ≡ 0.
(II) Association/dissociation of *Bb*-*cC* (association constant *k*_2_; dissociation constant *l*_2_):
  (20) b-c bB+cC⇌l2k2BbcC.
(III) Association/dissociation of *Cc*-*cC* (association constant *k*_3_; dissociation constant *l*_3_):
  (21) c-c cC+cC⇌l3k3CccC.

For reactions (I)–(III), there are twelve molecules in the PWN in Figure 3: *a*, *b*, *c*, *ab*, *bc*, *cc*, *abc*, *bcc*, *abcc*, *bccb*, *abccb*, and *abccba*. We denote their concentrations as *X*_1_ = [*a*], *X*_2_ = [*b*], *X*_3_ = [*c*], and so on. The three association/dissociation groups are presented in Tables 1, 2, and 3. We make the following basic assumptions:

(A1) For the association/dissociation of the modified molecules *Aa* and *bB*, we use the same association/dissociation constants as those used for the elementary reaction *a*-*b*. The same is assumed for *Bb*-*cC* and *Cc*-*cC*.
(A2) The initial concentrations for *X*_4_,…, *X*_12_ are all 0: *X*_*i*_(0) = 0, *i* = 4,…, 12.

In order to obtain the correct ODEs, it is necessary to extract the appropriate* doubling rules* for the reaction coefficients in the PWN. We will explain it in the next subsection.

### 3.2. Pathway Network Dynamics and Doubling Rules

From Figure 3 and Tables 1–3, we can write down the following twelve second-order nonlinear ODEs:

(E1) dX1dt=−k1X1X2+X5+X8+2X10+X11.

(E2) dX2dt=−k1X1X2−k2X2X3+2X6+X8+X9+l2X5+X8+2X10+X11.

(E3) dX3dt=−k2X3X2+X4−2k3X3X3+X5+X7+l2X5+X7+l32X6+X8+X9.

(E4) dX4dt=k1X1X2−k2X4X3+2X6+X8+X9+l2X7+X9+X11+2X12.

(E5) dX5dt=−k1X1X5+k2X2X3−2k3X5X3+X5+X7−l2X5+l3X8+2X10+X11.

(E6) dX6dt=k3X32−2k2X6X2+X4+l2X8+X9−l3X6.

(E7) dX7dt=k1X1X5+k2X3X4−2k3X7X3+X5+X7−l2X7+l3X9+X11+2X12.

(E8) dX8dt=−k1X1X8+2k2X2X6−k2X8X2+X4+2k3X3X5+l2−X8+2X10+X11−l3X8.

(E9) dX9dt=k1X1X8−k2X9X2+X4+2k2X4X6+2k3X3X7+l2−X9+X11+2X12−l3X9.

(E10) dX10dt=−2k1X1X10+k2X2X8+k3X52−2l2X10−l3X10.

(E11) dX11dt=k1X12X10−X11+k2X2X9+k2X4X8+2k3X5X7−2l2X11−l3X11.

(E12) dX12dt=k1X1X11+k2X4X9+k3X72−2l2X12−l3X12.

Note that ODEs E1–E12 incorporate doubling rules. The coefficient of 2 on

**Figure 4:**
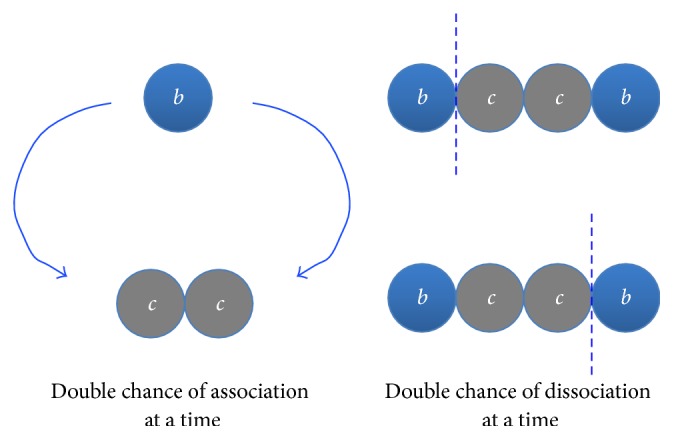
Doubling rules in the association/dissociation of *b*- and -*cC*.

1. *k*_3_*X*_3_^2^ in E3,
2. *k*_3_*X*_5_^2^ in E5,
3. *k*_3_*X*_7_^2^ in E7

corresponds to the double rate of consumption/reproduction in reaction (ii). See Figure 4. The coefficient of 2 on

1. *k*_3_*X*_3_(*X*_5_ + *X*_7_) in E3,
2. *k*_3_*X*_5_(*X*_3_ + *X*_7_) in E5,
3. *k*_3_*X*_7_(*X*_3_ + *X*_5_) in E7,
4. *k*_3_*X*_3_*X*_5_ in E8,
5. *k*_3_*X*_3_*X*_7_ in E9,
6. *k*_3_*X*_5_*X*_7_ in E11,

which corresponds to *Cc*-*cC* association or dissociation reactions in Table 3, will be explained in Section 4.3. The remaining coefficients of 2 in the ODEs are due to the double chance of association/dissociation in reaction (iv), as discussed in Section 2.

What is the rationale for the above doubling rules? It is the ingeniously well-formed MCLs and reaction laws, which are shown in the next section.

## 4. Analyzing the PWN Dynamics

### 4.1. Kinetics of Those Reactions Containing *a*-*bB*

According to Table 1, the reactions related to *a*-*bB* are summarized as

(22) $$\begin{array}{c} X_{1}+\left(\;X_{2}+X_{5}+X_{8}+2X_{10}+X_{11}\;\right)\overset{k_{1}}{\underset{l_{1}\equiv 0}{⇌}}\\ \left(\;X_{4}+X_{7}+X_{9}+X_{11}+2X_{12}\;\right). \end{array}$$

Looking at *X*_1_ and the products (the right hand side) of this reaction, we infer from the flux balance of consumption-production that we may have

(23) $$\begin{array}{c} \frac{d\left(X_{1}+X_{4}+X_{7}+X_{9}+X_{11}+2X_{12}\right)}{dt}\equiv 0. \end{array}$$

By taking summation E1 + E4 + E7 + E9 + E11 + 2 × E12 of the ODEs, we find that (23) is indeed true. Here, *X*_12_(*abccba*) has a coefficient of 2 because the decrease in *X*_1_ is equal to the increase in (*X*_4_ + *X*_7_ + *X*_9_ + *X*_11_ + 2*X*_12_), since the complex *abccba* consumes two *a* molecules, unlike the others: *ab*, *abc*, *abcc*, *abccb*. Equation (23) is an MCL of form (6a) or (17a). Equation (23) may be considered as an MCL for *a*_0_, since

(24) X1+X4+X7+X9+X11+2X12t≡const.≡X10=a0

by assumption (*A*2); thus, a local balance holds: *a*_0_ − *X*_1_(*t*)≡(*X*_4_ + *X*_7_ + *X*_9_ + *X*_11_ + 2*X*_12_)(*t*) for all *t* ≥ 0.

From (22), we also have

(25) $$\begin{array}{c} \frac{d\left(-X_{1}+X_{2}+X_{5}+X_{8}+2X_{10}+X_{11}\right)}{dt}\equiv 0, \end{array}$$

which may be considered to represent an MCL for *a*_0_ − *b*_0_, since

(26) −X1+X2+X5+X8+2X10+X11t≡const.≡−X10+X20=−a0+b0.

Here, *X*_10_ has a coefficient of 2 because the complex *bccb* has two *b*- sites for binding with *a*, which implies a double chance of association, as in reaction (iv). Equation (26) is an MCL of form (6b) or (17b).

Note that E1 and (25) suggest that *X*_1_ and (*X*_2_ + *X*_5_ + *X*_8_ + 2*X*_10_ + *X*_11_) form a pair of *a*-*bB* kinetics, (*E*_1_) and (*E*_2581011_), as displayed in Table 5. The two ODEs can be solved explicitly using the method for reaction (i) in Section 2; details are shown in Appendix A.1.

### 4.2. Kinetics of Those Reactions Containing *Bb*-*cC*

Next, we consider the kinetics of associations/dissociations with *Bb*-*cC* involved in the ODE system. As can be seen in Table 2, the reactions related to *Bb*-*cC* are summarized as

(27) $$\begin{array}{c} \left(\;X_{2}+X_{4}\;\right)+\left(\;X_{3}+2X_{6}+X_{8}+X_{9}\;\right)\overset{k_{2}}{\underset{l_{2}}{⇌}}\\ \left(\;X_{5}+X_{8}+2X_{10}+X_{11}\;\right)+\left(\;X_{7}+X_{9}+X_{11}+2X_{12}\;\right). \end{array}$$

In the manner similar to that used for the *Aa*-*bB* kinetics, we infer, from the flux balance of consumption-production, that we may have

(28) dX2+X5+X8+2X10+X11+X4+X7+X9+X11+2X12dt=0;

that is,

(29) dX2+X4+X5+X7+X8+X9+2X10+2X11+2X12dt=0.

By taking summation E1 + E4 + E7 + E9 + E11 + 2 × E12 of the ODEs, we find that (28) is indeed valid. Equation (28) is an MCL of form (6a) or (17a). Equstion (29) may be viewed as an MCL of *b*_0_, since

(30) X2+X4+X5+X7+X8+X9+2X10+2X11+2X12t≡const.≡X20=b0.

From (27), we also have

(31) $$\begin{array}{c} \frac{d\left(-X_{2}-X_{4}+X_{3}+2X_{6}+X_{8}+X_{9}\right)}{dt}=0, \end{array}$$

which is confirmed by taking the summation −E2 − E4 + E3 + 2 × E6 + E8 + E9 of the ODEs. This is an MCL of form (6b) or (17b). Equation (31) represents an MCL of *b*_0_ − *c*_0_, since

(32) −X2−X4+X3+2X6+X8+X9t≡const.≡−X20+X30=−b0+c0.

Here, *X*_6_ has a coefficient of 2 because it has two *c*- sites for binding with *a* or *ab*.

The MCL (31) implies that *ξ*_24_(*t*)≜(*X*_2_ + *X*_4_)(*t*) and *ξ*_3689_(*t*)≜(*X*_3_ + 2*X*_6_ + *X*_8_ + *X*_9_)(*t*) are governed by the same ODE, up to the initial values. From the ODEs E1–E12, it follows that the two variables form a pair of *Bb*-*cC* kinetics, (*E*_24_) and (*E*_3689_), as in Table 4. The variables *ξ*_24_(*t*) and *ξ*_3689_(*t*) can be solved explicitly; details are shown in Appendix A.2.

Now, we find that each of *X*_2_(*t*) and *X*_4_(*t*) can be solved explicitly, as well. This is because the right-hand side of E2 contains only known terms, except for *X*_2_:

E2′ dX2dt=−k1X1t+k2ξ24t+l2−k2b0−c0X2t+l2X1t−a0+b0,

by (26) and (32). Hence *X*_2_(*t*) can be calculated by the method of variation of coefficients. See Appendix A.3. In this way, *X*_4_(*t*) can be obtained by *X*_4_(*t*) = *ξ*_24_(*t*) − *X*_2_(*t*).

### 4.3. Kinetics of Those Reactions Containing *Cc*-*cC*

We finally consider the kinetics of associations/dissociations of *Cc*-*cC* involved in the ODE system. As shown in Table 2, the reactions related to *Cc*-*cC* can be summarized as

(33) X3+X5+X7+X3+X5+X7⇌l3k32X6+X8+X9+X8+2X10+X11+X9+X11+2X12.

In a manner similar to that used previously, we infer from the flux balance of consumption-production that

(34) $$\begin{array}{c} \frac{d\left(X_{3}+2X_{6}+X_{8}+X_{9}+X_{5}+X_{8}+2X_{10}+X_{11}+X_{7}+X_{9}+X_{11}+2X_{12}\right)}{dt}=0; \end{array}$$

that is,

(35) dX3+X5+2X6+X7+2X8+2X9+2X10+2X11+2X12dt=0.

By taking the summation E3 + E5 + 2 × E6 + E7 + 2 × E8 + 2 × E9 + 2 × E10 + 2 × E11 + 2 × E12 of the ODEs, we find that (35) is indeed valid. Equation (34) is an MCL of form (6a) or (17a). Equation (35) may be viewed as an MCL of *c*_0_, since

(36) X3+X5+2X6+X7+2X8+2X9+2X10+2X11+2X12t≡const.≡X30=c0.

From E3, E5, and E7, we have the ODE (*E*_357_), which is displayed in Table 5, and in which we have used (30) and (32). The coefficient of 2 on *k*_3_*X*_3_(*X*_3_ + *X*_5_ + *X*_7_)^2^ in (*E*_357_) is a result of the summation of the ODEs E3, E5, and E7. But then, where do the coefficients of 2 on *k*_3_(*X*_3_ + *X*_5_ + *X*_7_) in E3, on *k*_3_*X*_5_(*X*_3_ + *X*_5_ + *X*_7_) in E5, and on *k*_3_*X*_7_(*X*_3_ + *X*_5_ + *X*_7_) in E7 come from? They stem from the following stoichiometry. For those reactions in Table 3 that correspond to the reaction (ii) in Section 2,

(37) X3c+X3c⟷X6cc,X5bc+X5bc⟷X10bccb,X7abc+X7abc⟷X12abccba,

the reason is the same as in (12); that is, it is caused by the double rate of consumption/reproduction. Hence, we must count these three reactions with the association/dissociation coefficients on 2*k*_3_ and 2*l*_3_ in E3, E5, and E7. For the reaction *X*_3_(*c*) + *X*_5_(*bc*)↔*X*_8_(*bcc*) corresponding to reaction (iii), both

(38) X3c+X5bc⟷X8bcc,X5bc+X3c⟷X8bcc

must be counted. The first reaction in (38) is the one contained in E3, and the second reaction is in E5. In the first reaction, the* subject* is *X*_3_(*c*), and it associates with the* object X*_5_(*bc*). We will name this molecule *c* = *c*_1_. In the second reaction, the* subject* is *X*_5_(*bc*), and it associates with the* object X*_3_(*c*). We will name this molecule *c* = *c*_2_. In other words, a molecule of *c* (=*object*) is associated with a molecule of *bc* (=*subject*). In general, *c* = *c*_1_ and *c*_2_ are different molecules.

The first reaction must be counted as a reaction of *X*_3_(*c*). Also, seen as a reaction of *X*_5_(*bc*), the second reaction must also be counted. Both reactions take place at the same time. The association/dissociation coefficients are *k*_3_ and *l*_3_, respectively, for both reactions.

However, now consider, for example, how *X*_3_(*c*) is consumed doubly by the amount of *c*_1_ and *c*_2_. In Table 3, other combinations of this kind are

(39) X5bc+X7abc⟷X11abccb,X7abc+X5bc⟷X11abccb,X3c+X7abc⟷X9abcc,X7abc+X3c⟷X9abcc.

The contribution of the reactions in (38) and (39) are therefore doubled. Combining the doubling of the reactions in (38), we obtain the third reaction in Table 5, which causes the term −2*k*_3_(*X*_3_ + *X*_5_ + *X*_7_)^2^ in *E*_357_ and the ODEs E3, E5, and E7.

Finally, in considering *X*_3_(*c*), the dissociation term *l*_3_(*X*_8_ + *X*_9_) need not be doubled, since only one molecule of *c* is produced from a molecule of *bcc*: *bc*-*c*; also, this dissociation is not involved in E5.

By the above arguments for the doubling rules, we have obtained ingeniously well-formed MCLs and reaction laws, as shown in Table 4, respectively. This may suggest that our doubling rules are justified.

Also, the above argument of *Cc*-*cC* kinetics may imply the following: suppose that, for association of molecules, *p*_1_, *p*_2_,…, *p*_*n*_, say, some combinations of coupling *p*_*i*_-*p*_*j*_ (1 ≤ *i*, *j* ≤ *n*), are possible and others not. Then, it does not hold that

(40) dX1+⋯+Xndt=−2kX1+⋯+Xn2+dissociation  term

in general. However, if all of *p*_*i*_ has a common binding cite, that is, all *p*_*i*_ are of the form -*cC*, then we do have (40). In the latter case, the doubling rule holds for (*X*_1_ + ⋯+*X*_*n*_)* as a unit*, as if (*p*_1_, *p*_2_,…, *p*_*n*_) are a single molecule with binding cite -*c*, and the elementary doubling rule (12) holds with [*A*] in (12) replaced by (*X*_1_ + ⋯+*X*_*n*_).

### 4.4. Solutions to Remaining Variables

Solutions to the remaining variables can also be obtained. *X*_3_ and *X*_5_ can be found by the method of variation of parameters, as in the case of *X*_2_. *X*_7_ can be found by *X*_7_(*t*) = *ξ*_357_(*t*)−(*X*_3_ + *X*_5_)(*t*), where *ξ*_357_(*t*) is the solution to (*X*_3_ + *X*_5_ + *X*_7_)(*t*). By applying the solution *ξ*_357_ to E3, we obtain the solution to *X*_3_(*t*) as

E3′ dX3dt=−k2−l3ξ24+2k3−l2ξ357+l2+l3X3t+l2ξ357+l3ξ24−l3b0−c0

by (30) and (32); note that *ξ*_24_ and *ξ*_357_ have been obtained already. Similarly, *X*_6_, *X*_8_, and *X*_10_ can also be found by the method of variation of coefficient.

All of *X*_1_ to *X*_8_ and *X*_10_ have thus been obtained in principle. The remaining ones, *X*_9_, *X*_11_, and *X*_12_, can be obtained by solving

(41) X9t=ξ3689t−X3+2X6+X8t,X11t=X1t−X2+X5+X8+2X10t−a0+b0,X12t=12a0−X1+X4+X7+X9+X11t

(see (A.8), (26), and (24), resp.). The processes for obtaining these solutions are listed in Table 6.

### 4.5. Biomedical Implication

As described in Section 3.1, an appropriate amount of *X*_2_(*b*) (TIMP2) is necessary to obtain a substantial amount of the key molecule *X*_9_(*abcc*). Therefore, development and medication of such drugs that inhibit TIMP2 outside the cell membrane may be considered to be effective. Similarly, drugs that inhibit MT1-MMP inside the cell membrane might be considered to be useful as well.

**Figure 5:**
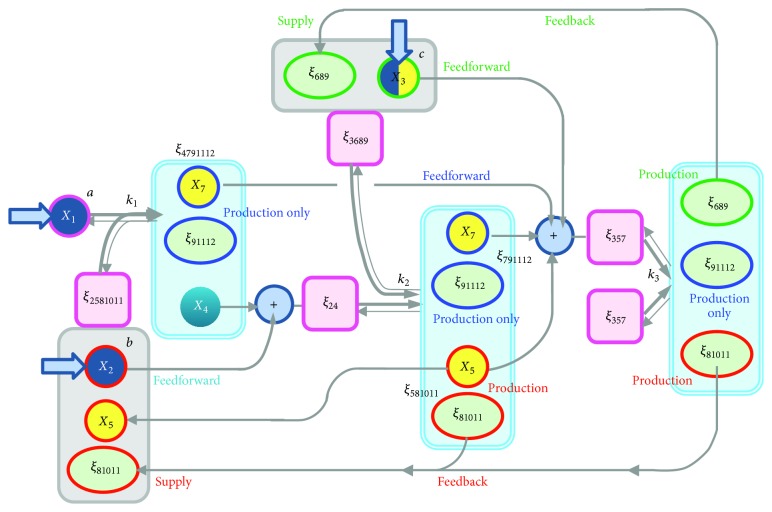
Looking at PWN with unit variables.

In addition, upon being clarified the model behaviors we notice the most important pathways in the PWN. They are three parts producing *ξ*_91112_ (association parts just before the three blue boxes in Figure 5). The PWN may be said to be such a device that continues to produce *ξ*_91112_, as explained in the following. In fact, the key molecule *X*_9_(*abcc*) is thus produced, through *ξ*_91112_, continuously as far as possible.

The initial concentration *a*_0_ is* dispersed* in the network, to *X*_4_(*ab*), *X*_7_(*abc*), *X*_9_(*abcc*), *X*_11_(*abccb*), and *X*_12_(*abccba*), according to the first MCL in Table 4. Likewise, *b*_0_ and *c*_0_ are* dispersed* in the network, according to latter two MCLs in Table 4, respectively.

The units of variables correspond to both the MCLs in Table 4 and reactions in Table 5. For example, the first MCL in Table 4 means that the consumption of *X*_1_ and production of *X*_4_(*ab*) + *X*_7_(*abc*) + *X*_9_(*abcc*) + *X*_11_(*abccb*) + 2*X*_12_ are of the same rate in the reaction (*a*, *b*) in Table 5.

Thus, the initial “mass” continues to exist in the PWN with its components changing to dimer, trimer, tetramer,…: at every moment, such dimer, trimer,…, and hexamer* contained in the unit variables* coexist. This is true for all the initial masses *a*_0_, *b*_0_, and *c*_0_. By this, in the* reactions by unit variables*,

1. *X*_1_ versus *ξ*_2581011_ = *X*_2_ + *X*_5_ + *X*_8_ + 2*X*_10_ + *X*_11_,
2. *ξ*_24_ = *X*_2_ + *X*_4_ versus *ξ*_3689_ = *X*_3_ + 2*X*_6_ + *X*_8_ + *X*_9_,
3. *ξ*_357_ = *X*_3_ + *X*_5_ + *X*_7_ versus *ξ*_357_ = *X*_3_ + *X*_5_ + *X*_7_,

*all possible intermediates of different level oligomers*, towards *X*_9_, are produced simultaneously at every moment. That is, the PWN system is settled so as to continue to produce *X*_9_ as much as possible at every moment.

In addition, the node that produces *X*_9_(*abcc*) is not single. There are three such nodes in the PWN, each in reaction of (*a*, *b*), (*b*, *c*), and (*c*, *c*). As in Figure 5, *X*_9_ is produced through the production of *ξ*_91112_ = *X*_9_ + *X*_11_ + 2*X*_12_. Here, this *ξ*_91112_ is only produced and no more reused by feedback or additional reactions.

Therefore, we may say that the PWN is such a system that produces *X*_9_ (through *ξ*_91112_) in the triple way (three production nodes) and continues the production as much as possible. This kind of consideration has become possible because the PWN model is analyzed through the unit variables.

### 4.6. On the Simulation Results

**Figure 6:**
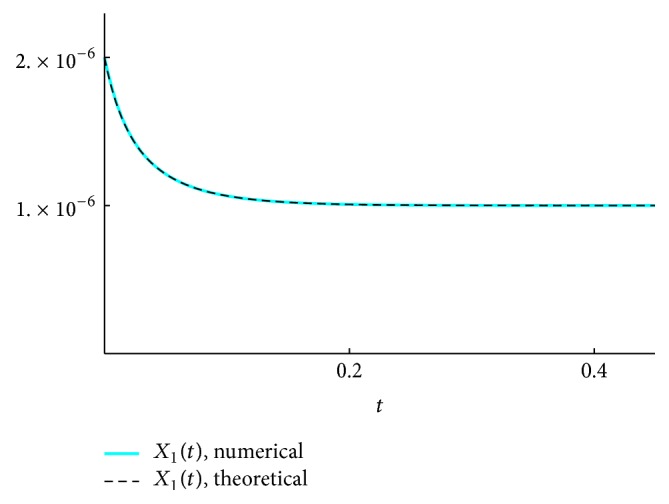
*X*_1_: simulation result and theoretical solution.

**Figure 7:**
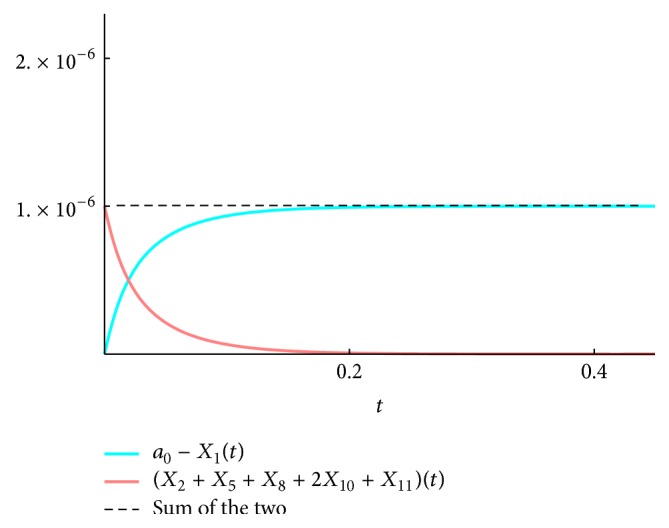
*ξ*_2581011_: simulation result and theoretical solution and MCL (26).

**Figure 8:**
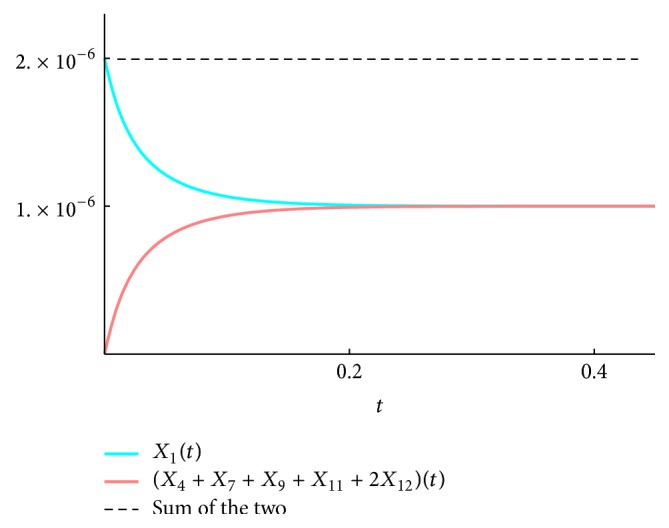
*ξ*_4791112_: simulation result and theoretical solution and MCL (24).

**Figure 9:**
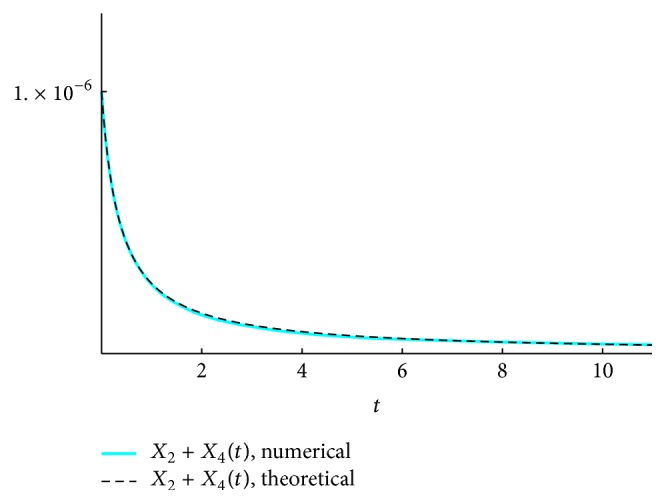
*ξ*_24_: simulation result and theoretical solution.

**Figure 10:**
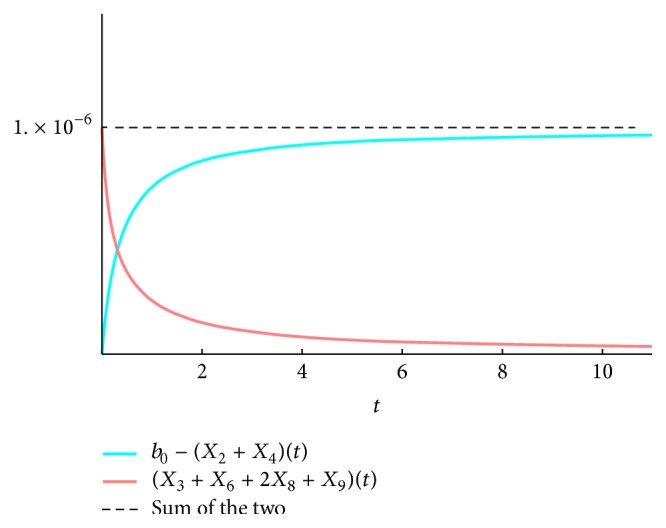
*ξ*_3689_: simulation result and theoretical solution and MCL (32).

**Figure 11:**
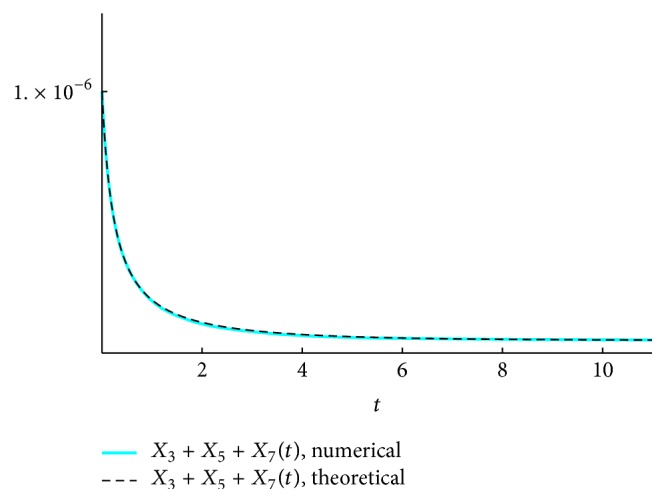
*ξ*_357_: simulation result and theoretical solution.

**Figure 12:**
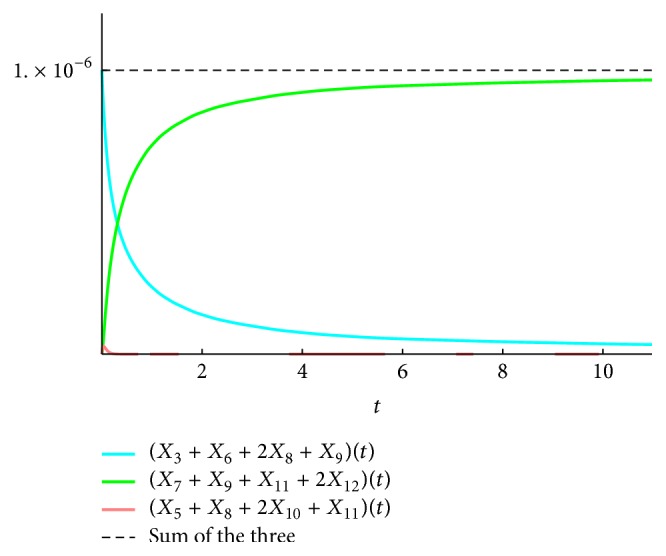
*ξ*_3689_ + *ξ*_581011_ + *ξ*_791112_: simulation result and theoretical solution and MCL (36).

We present the time course plots of the theoretical and numerical solutions in Figures 6–12. The theoretical solutions above are in good agreement with our simulation results. In Figures 7, 8, 10, and 12, the MCLs (26), (24), (32), and (34), respectively, are confirmed; the solutions are also confirmed. The values of reaction constants and initial values used in the simulations are listed in Table 7. The reaction constants are the same as those used in Watanabe et al. [4], while the initial values are set as follows: the initial values used in [4] are *a*_0_ = *b*_0_ = *c*_0_ = 1.0 × 10^−7^ [M] based on experimental measurement; if the initial values are adopted as they stand, then the simulation causes a time-delay; that is, the responses become much slower. Therefore, for the sake of convenience in simulation, we take the initial values with the order of 10^−6^. In addition, in order to show the relationship of the behaviors of solutions and initial values in a more effective way, we set as *a*_0_ = 2.0 × 10^−6^ [M]. For example, in Figure 6, we can see that *X*_1_(*∞*) = *a*_0_ − *b*_0_ indeed (see (A.1)). Also, in Figure 8, the MCL can be seen visually.

It should be noted that the simulation results obtained in Watanabe et al. [4] and hence in this paper are based on actual cancer data. The study in this paper provides the exact solutions to the ODE model and hence a mathematically rigorous foundation is added to the simulation results.

Concerning the question that if there is a threshold of the key molecule concentration that leads to ECM degradation, we do not know the exact threshold at least presently. It may be considered that, according to the amount of the key molecule, ECM degradation is promoted weakly or strongly. However, as for the unit variable *ξ*_3689_, it is indicated in Watanabe et al. [4] that, at around *b*_0_ = 100 [nM], transient peak of *ξ*_3689_ becomes maximal and the ECM degradation is promoted thereby the most. See Figures S3, S4, and S5 in [4].

**Figure 13:**
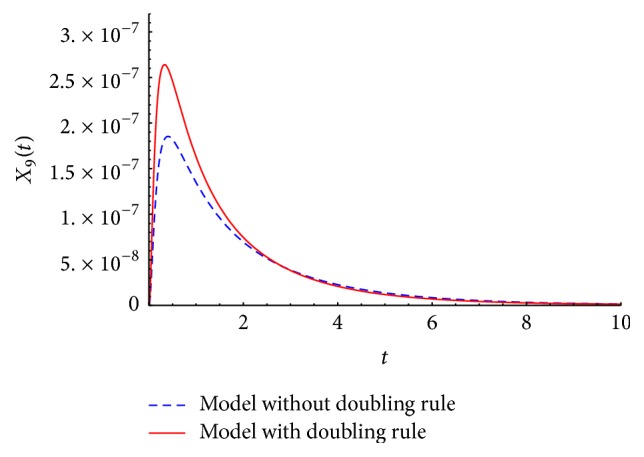
Time course of *X*_9_(*t*): comparison of the models with or without the doubling rule.

Figure 13 presents the time courses of a simulation result of the key molecule *X*_9_ for the model with and without the doubling rule. Here, the initial values of the concentration are *a*_0_ = 2 × 10^−6^ [M], *b*_0_ = 2 × 10^−6^ [M], and *c*_0_ = 1.8 × 10^−6^ [M]. By modifying the model with the doubling rule, the peak at about *t* = 0.25 was increased by approximately 1.5 times.

Actually, the authors did not begin with the idea of the doubling rules in Section 3.2. The only doubling rule we had exploited was the one corresponding to the general basic model in (12). Thus the only coefficients of 2 were on *k*_3_*X*_3_^2^ and *l*_3_*X*_6_ in E3, *k*_3_*X*_5_^2^ and *l*_3_*X*_10_ in E5, *k*_3_*X*_7_^2^ and *l*_3_*X*_12_ in E7, and *l*_2_*X*_11_ in E11. The solutions to this first version of the model and the modified version in E1–E12 are different, but they have the same initial and asymptotic behaviors. In Figure 13, we show as an example the time series of the key molecule *X*_9_(*t*).

**Figure 14:**
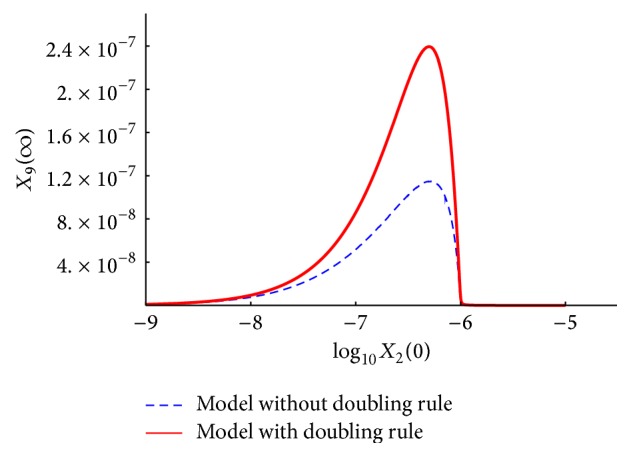
*X*_9_(*∞*) versus *X*_2_(0): comparison of the models with or without the doubling rule.

Figure 14 shows a plot of *X*_9_(*∞*) versus *X*_2_(0). As described in Section 3.1, the key molecule of the ECM degradation is *abcc* (concentration *X*_9_), and the amount of *abcc* that is produced is influenced primarily by *b*_0_. If there is too much or too little *b*_0_, an insignificant amount of *abcc* will be produced. As shown in the figure, in both models, *X*_9_(*∞*) has a peak at around *b*_0_ = 5.1 × 10^−7^ [M]. The peak increases when the model was modified.

## 5. Concluding Remarks

We presented a method for solving a nonlinear ODE system from cell biology. With the setting in this paper, the ODEs become a completely integrable system so that they can be solved explicitly. The key idea was to use the MCL to obtain linear relations of the ODE variables that would be valid for all *t* ≥ 0. The previous analysis of such ODE systems was primarily based on the balance of flux at equilibrium (*t* = *∞*) or by computer simulation (for *t* < *∞*). The theoretical solutions to the ODEs are in complete agreement with those obtained by simulation.

We gained several new insights in analyzing and modeling the PWNs: in the MALs, a coefficient of 2 must be attached sometimes to appropriate molecules. Determining all of the doubling laws is crucial for obtaining an integrable system of ODEs. We have elucidated some situations in which the doubling law is required. We also note that the total PWN can be grouped into several local PWNs in which local linear relationships hold. This may suggest a way to obtain an appropriate view of the composition of network motifs in systems biology.

In a future study, we will try to determine a sufficient condition such that the given ODE system is completely integrable. It may be desirable to clarify under which conditions the ODE system framework is sufficiently “close” to a realistic PDE system; this would be useful for the development of therapies or drugs.

## Figures and Tables

**Table 1:** List of associations with *a*-*bB*.

Association with *a*-*bB*		Assoc. const.	Dissoc. const.
*X*_1_(*a*)	+*X*_2_(*b*)⟶*X*_4_(*ab*)	*k*_1_	*l*_1_ = 0
	+*X*_5_(*bc*)⟶*X*_7_(*abc*)	*k*_1_	
	+*X*_8_(*bcc*)⟶*X*_9_(*abcc*)	*k*_1_	
	+*X*_10_(*bccb*)⟶*X*_11_(*abccb*)	2*k*_1_	
	+*X*_11_(*abccb*)⟶*X*_12_(*abccba*)	*k*_1_	

**Table 2:** List of associations/dissociations with *Bb*-*cC*.

Association/dissociation with *Bb*-*cC*		Assoc. const.	Dissoc. const.
*X*_2_(*b*)	+*X*_3_(*c*)⟷*X*_5_(*bc*)	*k*_2_	*l*_2_
	+*X*_6_(*cc*)⟷*X*_8_(*bcc*)	2*k*_2_	*l*_2_
	+*X*_8_(*bcc*)⟷*X*_10_(*bccb*)	*k*_2_	2*l*_2_
	+*X*_9_(*abcc*)⟷*X*_11_(*abccb*)	*k*_2_	*l*_2_

*X*_4_(*ab*)	+*X*_3_(*c*)⟷*X*_7_(*abc*)	*k*_2_	*l*_2_
	+*X*_6_(*cc*)⟷*X*_9_(*abcc*)	2*k*_2_	*l*_2_
	+*X*_8_(*bcc*)⟷*X*_11_(*abccb*)	*k*_2_	*l*_2_
	+*X*_9_(*abcc*)⟷*X*_12_(*abccba*)	*k*_2_	2*l*_2_

**Table 3:** List of associations/dissociations with *Cc*-*cC*.

Association/dissociation with *Cc*-*cC*		Assoc. const.	Dissoc. const.
*X*_3_(*c*)	+*X*_3_(*c*)⟷*X*_6_(*cc*)	2*k*_3_	2*l*_3_
	+*X*_5_(*bc*)⟷*X*_8_(*bcc*)	*k*_3_	*l*_3_
	+*X*_7_(*abc*)⟷*X*_9_(*abcc*)	*k*_3_	*l*_3_

*X*_5_(*bc*)	+*X*_3_(*c*)⟷*X*_8_(*bcc*)	*k*_3_	*l*_3_
	+*X*_5_(*bc*)⟷*X*_10_(*bccb*)	2*k*_3_	2*l*_3_
	+*X*_7_(*abc*)⟷*X*_11_(*abccb*)	*k*_3_	*l*_3_

*X*_7_(*abc*)	+*X*_3_(*c*)⟷*X*_9_(*abcc*)	*k*_3_	*l*_3_
	+*X*_5_(*bc*)⟷*X*_11_(*abccb*)	*k*_3_	*l*_3_
	+*X*_7_(*abc*)⟷*X*_12_(*abccba*)	2*k*_3_	2*l*_3_

**Table 4:** Mass conservation laws of *a*, *b*, and *c*.

Items	MCL
*a*_0_	$\frac{d\left(X_{1}+X_{4}+X_{7}+X_{9}+X_{11}+2X_{12}\right)}{dt}\equiv 0$
*b*_0_	$\frac{d\left(X_{2}+X_{4}+X_{5}+X_{7}+X_{8}+X_{9}+2X_{10}+2X_{11}+2X_{12}\right)}{dt}\equiv 0$
*c*_0_	$\frac{d\left(X_{3}+X_{5}+2X_{6}+X_{7}+2X_{8}+2X_{9}+2X_{10}+2X_{11}+2X_{12}\right)}{dt}\equiv 0$

**Table 5:** Reaction laws of (*a*, *b*), (*b*, *c*), and (*c*, *c*).

(*a*, *b*): $X_{1}+\left(X_{2}+X_{5}+X_{8}+2X_{10}+X_{11}\right)\overset{k_{1}}{\underset{l_{1}\equiv 0}{⇌}}\left(X_{4}+X_{7}+X_{9}+X_{11}+2X_{12}\right)⟺$	
	$\left\{\begin{array}{cc} \left(E_{1}\right) & \frac{dX_{1}}{dt}=-k_{1}X_{1}\left(X_{2}+X_{5}+X_{8}+2X_{10}+X_{11}\right)\\ \left(E_{2581011}\right) & \frac{d\left(X_{2}+X_{5}+X_{8}+2X_{10}+X_{11}\right)}{dt}=-k_{1}X_{1}\left(X_{2}+X_{5}+X_{8}+2X_{10}+X_{11}\right). \end{array}\right.$

(*b*, *c*): $\left(X_{2}+X_{4}\right)+\left(X_{3}+2X_{6}+X_{8}+X_{9}\right)\overset{k_{2}}{\underset{l_{2}}{⇌}}\left(X_{5}+X_{7}+X_{8}+X_{9}+2X_{10}+2X_{11}+2X_{12}\right)⟺$	
	$\left\{\begin{array}{cc} \left(E_{24}\right) & \frac{d\left(X_{2}+X_{4}\right)}{dt}=-k_{2}\left(X_{2}+X_{4}\right)\left(X_{3}+2X_{6}+X_{8}+X_{9}\right)+l_{2}\left(X_{5}+X_{7}+X_{8}+X_{9}+2X_{10}+2X_{11}+2X_{12}\right)\\ \left(E_{3689}\right) & \frac{d\left(X_{3}+2X_{6}+X_{8}+X_{9}\right)}{dt}=-k_{2}\left(X_{2}+X_{4}\right)\left(X_{3}+2X_{6}+X_{8}+X_{9}\right)+l_{2}\left(X_{5}+X_{7}+X_{8}+X_{9}+2X_{10}+2X_{11}+2X_{12}\right). \end{array}\right.$

(*c*, *c*): $\left(X_{3}+X_{5}+X_{7}\right)+\left(X_{3}+X_{5}+X_{7}\right)\overset{k_{3}}{\underset{l_{3}}{⇌}}\left(2X_{6}+X_{8}+X_{9}+X_{8}+2X_{10}+X_{11}+X_{9}+X_{11}+2X_{12}\right)⟺$	
	$(E_{357})\;\frac{d\left(X_{3}+X_{5}+X_{7}\right)}{dt}=-2k_{3}{\left(X_{3}+X_{5}+X_{7}\right)}^{2}+2l_{3}\left(X_{6}+X_{8}+X_{9}+X_{10}+X_{11}+X_{12}\right)$

**Table 6:** Processes of obtaining solutions *X*_1_–*X*_12_.

Variable	Method
*X*_1_	Single Riccati equation
*ξ*_2581011_	A companion of *X*_1_
*ξ*_24_	Single Riccati equation
*ξ*_3689_	A companion of *ξ*_24_
*X*_2_	Method of variation of coefficient
*X*_4_	*X*_4_ = *ξ*_24_ − *X*_2_
*ξ*_357_	Single Riccati equation
*X*_3_, *X*_5_	Method of variation of coefficient
*X*_7_	*X*_7_ = *ξ*_357_ − (*X*_3_ + *X*_5_)
*X*_6_, *X*_8_, *X*_10_	Method of variation of coefficient
*X*_9_, *X*_11_, *X*_12_	by (41)

**Table 7:** Initial values and reaction constants used in the simulation.

Variables	Values
*a*_0_	2.0 × 10^−6^ [M]
*b*_0_	1.0 × 10^−6^ [M]
*c*_0_	1.0 × 10^−6^ [M]

*k*_1_	2.1 × 10^7^ [M^−1^ s^−1^]
*l*_1_	0 [M^−1^ s^−1^]

*k*_2_	2.74 × 10^6^ [M^−1^ s^−1^]
*l*_2_	2.0 × 10^−4^ [M^−1^ s^−1^]

*k*_3_	2.0 × 10^6^ [M^−1^ s^−1^]
*l*_3_	1.0 × 10^−2^ [M^−1^ s^−1^]

## Appendix

### A. Explicit Solutions to the Unit Variables

The solutions to *X*_1_–*X*_12_ and related materials are presented as follows.

#### A.1. Solution to *X*_1_: Single Riccati Equation

Theorem A.1 A.1. One has the following solution to (*E*_1_):

(A.1) $$\begin{array}{c} X_{1}\left(\;t\;\right)=\left\{\begin{array}{cc} \frac{a_{0}\left(a_{0}-b_{0}\right)}{a_{0}-b_{0}e^{-k_{1}\left(a_{0}-b_{0}\right)t}}, & \left(a_{0}>b_{0}\right),\\ \frac{b_{0}}{1+k_{1}b_{0}t}, & \left(a_{0}=b_{0}\right),\\ \frac{a_{0}\left(b_{0}-a_{0}\right)e^{-k_{1}\left(b_{0}-a_{0}\right)t}}{b_{0}-a_{0}e^{-k_{1}\left(b_{0}-a_{0}\right)t}}, & \left(a_{0}<b_{0}\right). \end{array}\right. \end{array}$$

Corollary A.2 A.2. 
*X*_1_(*t*) is decreasing monotonically:

(A.2) X10=a0↘X1∞=a0−b0,a0>b0,0,a0≤b0.

Thus *X*_1_(*t*) is nonnegative as well. Let us denote *ξ*_2581011_(*t*)≜(*X*_2_ + *X*_5_ + *X*_8_ + 2*X*_10_ + *X*_11_)(*t*).

Corollary A.3 A.3. One has the following solution to (*E*_2581011_):

(A.3) $$\begin{array}{c} \xi_{2581011}\left(\;t\;\right)=\left\{\begin{array}{cc} \frac{b_{0}\left(a_{0}-b_{0}\right)e^{-k_{1}\left(a_{0}-b_{0}\right)t}}{a_{0}-b_{0}e^{-k_{1}\left(a_{0}-b_{0}\right)t}}, & \left(a_{0}>b_{0}\right),\\ \frac{b_{0}}{1+k_{1}b_{0}t}, & \left(a_{0}=b_{0}\right),\\ \frac{b_{0}\left(b_{0}-a_{0}\right)}{b_{0}-a_{0}e^{-k_{1}\left(b_{0}-a_{0}\right)t}}, & \left(a_{0}<b_{0}\right). \end{array}\right. \end{array}$$

Thus *ξ*_2581011_(*t*) is decreasing monotonically:

(A.4) ξ25810110=b0↘ξ2581011∞=0,a0≥b0,b0−a0,a0<b0

and is nonnegative.

#### A.2. Solution to *X*_2_ + *X*_4_: Single Riccati Equation

Theorem A.4 A.4. One has the following solution to (*E*_24_):

(A.5) ξ24t≜X2+X4t=z1−C24z2e−k2z1−z2t1−C24e−k2z1−z2t,C24=b0−z1b0−z2,

where

(A.6) z1,z2=k2b0−c0−l2±k2b0−c0−l22+4k2l2b02k2.

We note that *b*_0_ − *c*_0_ > *z*_1_ > 0 > *z*_2_. Since (*X*_2_ + *X*_4_)(*t*) = *z*_2_ + (*z*_1_ − *z*_2_)/(1 − *C*_24_*e*^−*k*_2_(*z*_1_−*z*_2_)*t*^), we have the following.

Corollary A.5 A.5. 
*ξ*_24_(*t*) is decreasing monotonically:

(A.7) ξ240=b0↘ξ24∞=z1.

Thus *ξ*_24_(*t*) is nonnegative as well.

Corollary A.6 A.6. One has the following solution to (*E*_3689_):

(A.8) ξ3689tX3+2X6+X8+X9t=z1−C24z2e−k2z1−z2t1−C24e−k2z1−z2t−b0+c0,t≥0.

Thus *ξ*_3689_(*t*) is decreasing monotonically:

(A.9) ξ36890=c0↘ξ3689∞=z1−b0+c0

and is nonnegative.

The solutions *ξ*_24_(*t*) and *ξ*_3689_(*t*) are thus obtained irrespective of *b*_0_ > *c*_0_ or *b*_0_ < *c*_0_. Each of the solutions to *X*_2_(*t*) and *X*_4_(*t*), however, does depend on *a*_0_ > *b*_0_, *a*_0_ = *b*_0_ or *a*_0_ < *b*_0_ as seen in the following.

#### A.3. Solution to *X*_2_ and Thus *X*_4_: Method of Variation of Constant

Let *E*_1_ = *e*^−*k*_1_(*b*_0_−*a*_0_)*t*^, *E*_2_ = *e*^−*k*_2_(*z*_1_−*z*_2_)*t*^, and *E*_3_ = *e*^−(*k*_2_*z*_1_+*l*_2_)*t*^.

Theorem A.7 A.7. One has

(A.10) X2t=C1,1a0−b0E1E31−C1,1E11−C24E21−C24+l21−E3k2ζ1+l2−C241−E2E3k2z1−z2+ζ1+l2,a0>b0,b01+k1b0t1−C24E21−C24E3+l2C241−E31+k1b0tκ2−1−E2k2z1−z2−ζ1−l2−1−E21+k1b0t−1k2z1−z2−ζ1−l22,a0=b0,b0−a01−C1,3E11−C24E21−C24E3+l21−E3k2z1+l2+E3−E2k2z2+l2,a0<b0,

respectively. Here *κ*_2_ > 0 is given by

(A.11) κ2=ηk2ζ1+l2+ηk2z1−z2−ζ1−l2,with  ηx=x−1+k1b0x−2.

The solutions turn out to be nonnegative in either of the three cases. Now *X*_4_ is given by *X*_4_(*t*) = *ξ*_24_(*t*) − *X*_2_(*t*), although we do not write down the solution here because it is complicated like *X*_2_.

#### A.4. Solution to *X*_3_ + *X*_5_ + *X*_7_: Single Riccati Equation

The solution to (*E*_357_) is obtained, similarly as *ξ*_24_.

(A.12) ξ357tX3+X5+X7t=η1−η2C357e−2k3η1−η2t1−C357e−2k3η1−η2t,C357=c0−η1c0−η2,

where

(A.13) $$\begin{array}{c} \eta_{1},\eta_{2}=\frac{-l_{3}\pm \sqrt{l_{3}^{2}+8k_{3}l_{3}c_{0}}}{4k_{3}}, \end{array}$$

respectively. Properties of positivity and monotone decreasing of *ξ*_357_ are valid as well.

The solutions to other variables, *X*_3_, *X*_5_, *X*_7_, *X*_6_, *X*_8_, *X*_10_, *X*_9_, *X*_11_, and *X*_12_, are too complicated to write down and omitted.
